# Teaching case 3-2019: Are nuclear clefts or invaginations the niche of intranuclear inclusions in FTLD-TDP? 

**DOI:** 10.5414/NP301202

**Published:** 2019-04-26

**Authors:** Laura Molina-Porcel, Esther Pérez-Navarro, Marta García-Forn, David Westaway, Martí Colom-Cadena, Ellen Gelpi

**Affiliations:** 1 Neurological Tissue Bank of the Biobanc-Hospital Clinic-IDIBAPS, Institut d’Investigacions Biomèdiques August Pi I Sunyer,; 2 Emili Mira Center, Institute of Neuropsychiatry and Addictions (INAD),; 3 Department of Biomedical Sciences, Faculty of Medicine and Health Sciences, University of Barcelona,; 4 Institut d’Investigacions Biomèdiques August Pi i Sunyer (IDIBAPS),; 5 Centro de Investigación Biomédica en Red sobre Enfermedades Neurodegenerativas (CIBERNED), Barcelona, Spain,; 6 Division of Neurology, Department of Medicine Centre for Prions and Protein Folding Diseases Neuroscience and Mental Health Institute Faculty of Medicine & Dentistry, University of Alberta, AB, Canada,; 7 UK Dementia Research Institute and Centre for Discovery Brain Sciences, The University of Edinburgh, Edinburgh, Scotland, UK,; 8 Institute of Neurology, Medical University of Vienna, Allgemeines Krankenhaus der Stadt Wien, Vienna, Austria

**Keywords:** intranuclear inclusions, nuclear cleft, nuclear invagination, FTLD-TDP, neurodegeneration, Lamin B1

## Abstract

No abstract available.

Intranuclear inclusions, in addition to intracytoplasmic inclusions, are frequent findings in some sporadic and/or genetic neurodegenerative diseases [1]. TDP-43 and FUS are nuclear proteins with multiple functions in mRNA processing, which have been involved in amyotrophic lateral sclerosis (ALS) and frontotemporal lobar degeneration (FTLD) [2, 3, 4]. TDP-43 and FUS proteins shuttle between nucleus, and cytoplasm and defects in nucleocytoplasmic transport can contribute to these pathologies [5]. For example, in sporadic ALS and FTLD-FUS/FET (fused in sarcoma/Ewing sarcoma/TAF15) an accumulation of nuclear import protein Transportin 1 has been observed [6, 7]; and proteins involved in nucleocytoplasmic transport are major components of TDP-43 aggregates [8]. On the other hand, alterations of nuclear membrane morphology and formation of clefts or invaginations are a frequent finding in FTLD and other neurodegenerative diseases [9]. In FTLD-TDP, the lentiform morphology of some nuclear inclusions (Figure 1C) – also named cat-eye inclusion – is reminiscent of nuclear clefts or invaginations (Figure 1A, D). Similarly, the nuclear vermiform inclusions observed in aFTLD-U, a subtype of FTLD-FET, also mimic the morphology of nuclear clefts. This suggests that a fraction of TDP-43 and FUS proteins may accumulate within the nuclear clefts or invaginations and form a subtype of intranuclear inclusions characteristic of the disease, that may result into a cell-specific and disease-characteristic transcriptomic pattern. 

Nuclear clefts or invaginations are visible on HE-stained sections but are better identified by immunohistochemistry for the nuclear membrane protein lamin B1 (Figure 1B). However, TDP-43 cat-eye type nuclear inclusions do not necessarily contain nor are surrounded by the nuclear membrane on double immunofluorescence with lamin B1 in the case illustrated here (Figure 1E). This finding might suggest an alteration of the nuclear transport structure in neurons harboring apparently intranuclear inclusions. Alternatively, although defects in nucleocytoplasmic transport can contribute to this pathology, nuclear TDP-43 inclusions may develop independently of the nuclear membrane clefts. 

## Acknowledgment 

We wish to thank Brain Donors of the Neurological Tissue Bank of the Biobanc-Hospital Clinic-IDIBAPS and their families for their generosity, and Sara Charif and Veronica Santiago for their technical assistance. 

## Ethics 

All tissue samples were obtained according to Spanish Legislation. The informed consents for postmortem studies were signed by the patients or by their legal representatives in their name, as approved by local ethics committees and allowed by Spanish law. 

## Funding 

There was no specific funding for this study. 

## Conflict of interest 

The authors report no conflict of interest. 

**Figure 1.**
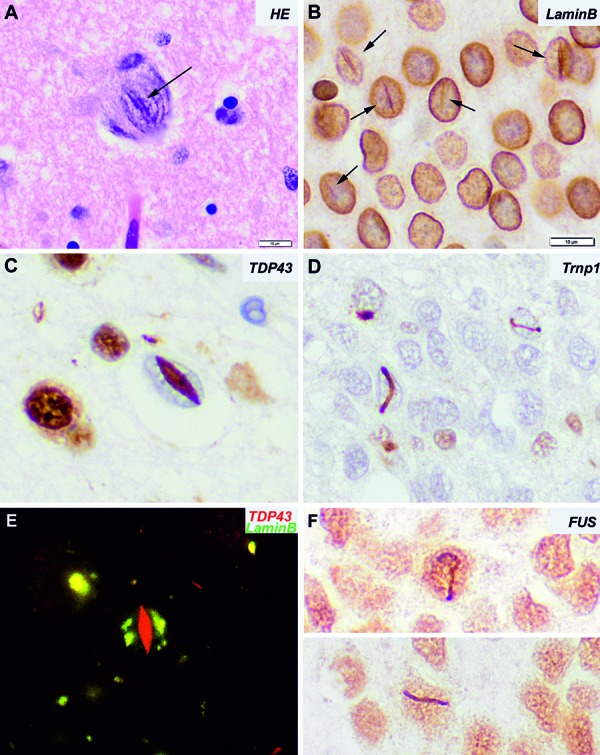
A: Hematoxylin-Eosin staining of a large neuron with a central basophilic cleft crossing the nucleus. B: Immunohistochemistry for lamin B1 nicely depicts the nuclear envelop and enhances the nuclear clefts. C: Cat-eye lentiform intranuclear inclusion in a patient with FTLD-TDP. D: Immunohistochemistry for Transportin 1 shows small lanceolated or vermiform intranuclear inclusions in granular neurons of the dentate gyrus of the hippocampus in a patient with aFTLD-U/ FTLD-FET. These inclusions are also immunoreactive for FUS (F). E: Double immunofluorescence for TDP43 and lamin B1 reveals that the cat-eye inclusion is not surrounded by the nuclear membrane. Instead, lamin B1 immunoreactivity shows an irregular distribution throughout the nucleus.
